# Supplementary figures and images for: A comparative study to optimize experimental conditions of pentylenetetrazol and pilocarpine-induced epilepsy in zebrafish larvae
Source: PLoS One. 2023 Jul 28;18(7):e0288904. doi: 10.1371/journal.pone.0288904 (PMC10381053; doi:10.1371/journal.pone.0288904)

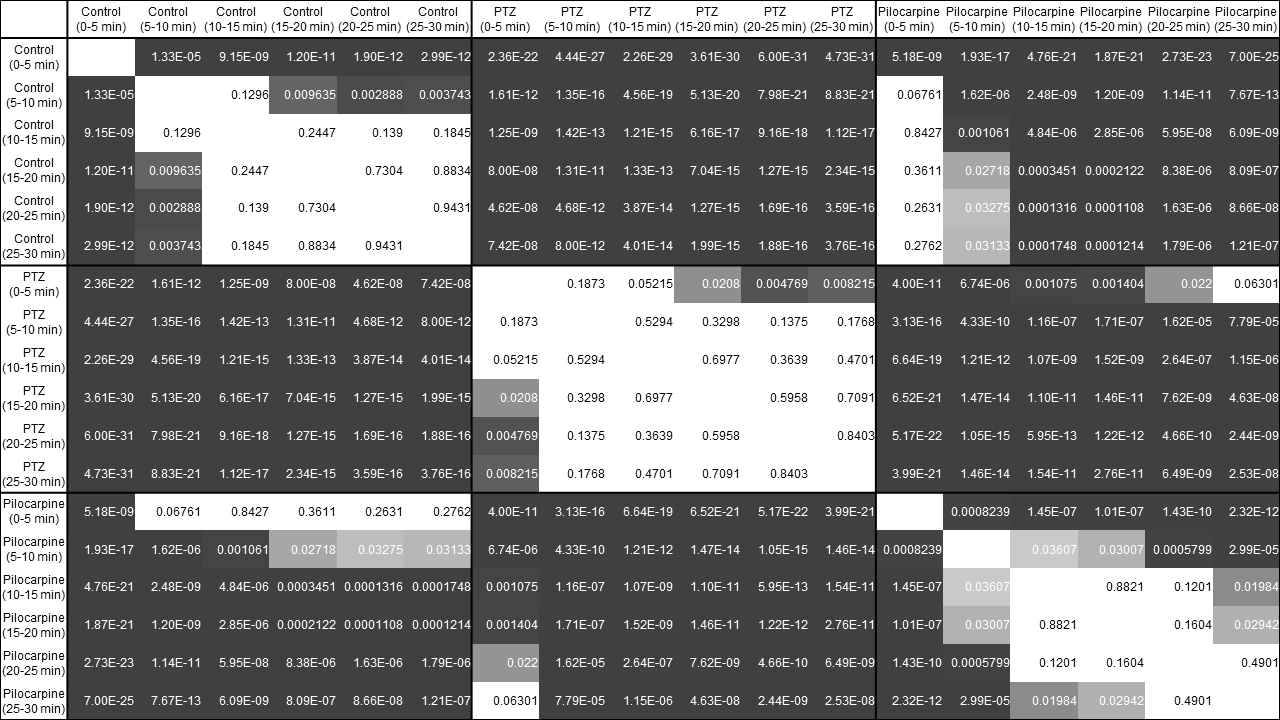

Supplement: S1 Fig — (TIF) [file pone.0288904.s001.tif]
