# Supplementary material for: A comparative study to optimize experimental conditions of pentylenetetrazol and pilocarpine-induced epilepsy in zebrafish larvae
Source: PLoS One. 2023 Jul 28;18(7):e0288904. doi: 10.1371/journal.pone.0288904 (PMC10381053; doi:10.1371/journal.pone.0288904)
Supplement: S1 Table — (DOCX) [file pone.0288904.s002.docx]

S1 Table

| Trial number - treatment | *r^2^* | *p* |
| --- | --- | --- |
| Trial 1 - Control | 0.9304 | 0.0001 |
| Trial 1 - PTZ | 0.5946 | 0.0001 |
| Trial 1 - Pilocarpine | 0.7420 | 0.0001 |
| Trial 2 - Control | 0.9105 | 0.0001 |
| Trial 2 - PTZ | 0.7597 | 0.0001 |
| Trial 2 - Pilocarpine | 0.9292 | 0.0001 |
| Trial 3 - Control | 0.7561 | 0.0001 |
| Trial 3 - PTZ | 0.5636 | 0.0001 |
| Trial 3 - Pilocarpine | 0.9742 | 0.0001 |
| Trial 4 - Control | 0.3730 | 0.0001 |
| Trial 4 - PTZ | 0.5506 | 0.0001 |
| Trial 4 - Pilocarpine | 0.9347 | 0.0001 |
| Trial 5 - Control | 0.4415 | 0.0001 |
| Trial 5 - PTZ | 0.7111 | 0.0001 |
| Trial 5 - Pilocarpine | 0.9213 | 0.0001 |
| Trial - 6 Control | 0.8231 | 0.0001 |
| Trial 6 - PTZ | 0.0735 | 0.0012 |
| Trial 6 - Pilocarpine | 0.9028 | 0.0001 |
| Trial 7 - Control | 0.8604 | 0.0001 |
| Trial 7 - PTZ | 0.0686 | 0.0072 |
| Trial 7 - Pilocarpine | 0.9804 | 0.0001 |
| Trial 8 - Control | 0.8703 | 0.0001 |
| Trial 8 - PTZ | 0.9869 | 0.0001 |
| Trial 8 - Pilocarpine | 0.7966 | 0.0001 |
| Trial 9 - Control | 0.6245 | 0.0001 |
| Trial 9 - PTZ | 0.9524 | 0.0001 |
| Trial 9 - Pilocarpine | 0.9072 | 0.0001 |
