# Supplementary material for: A comparative study to optimize experimental conditions of pentylenetetrazol and pilocarpine-induced epilepsy in zebrafish larvae
Source: PLoS One. 2023 Jul 28;18(7):e0288904. doi: 10.1371/journal.pone.0288904 (PMC10381053; doi:10.1371/journal.pone.0288904)
Supplement: Attachment — Submitted filename: Rebuttal letter.docx [file pone.0288904.s003.docx]

Dear Editors,

We would like to thank the reviewers for their time and very detailed and extremely useful comments on the manuscript. We have corrected the manuscript to address their concerns.

We believe that the manuscript is now suitable for publication in Plos One.

**Reviewer #1:**

*The zebrafish model is widely used to study the effects of anti-epileptic drugs. However, according to Szep et al, the methods used are often not sufficiently detailed, which leads to results discrepancies between laboratories. In their paper, they performed a series of experiments to optimise the zebrafish epilepsy model and found that the zebrafish larvae move significantly more in the light condition compared to the dark condition, independently of treatment with PTZ or pilocarpine. When examining the optimal observation time, they found that during the half-hour period, the first 5 minutes were found to be the most different from the others. They therefore recommend 30 minutes of drug pre-treatment followed by a 10-minute test in light conditions with a 5-minute accommodation time. For testing photolabile AED compound candidates, they recommend the pilocarpine test in the dark. Investigators in the field of epilepsy could benefit from such a refinement of the zebrafish model.*

*Abstract
The use of term “epileptics” is not the right term to use. If authors refer to PTZ or pilocarpine, it should be “chemoconvulsants” or “convulsants”.*

Thank you for your suggestion, we corrected the revised manuscript and used “convulsant” instead of “epileptics”.

*Materials and Methods
We left the fish in the solution for 30 for accommodation before starting the recording 10. Please correct. 30 s or min?*

Thank you for the comment, it is supposed to be 30 minutes, we corrected it.

*The Results section should be re-organized.* *Figure legends should not be placed in the text.*

Thank you for your suggestion, we reorganized as recommended. We removed the figure legends from the text, and placed them to the end, as suggested by the Submission Guidelines.

*Moreover, the results of statistical tests are provided in the first paragraph of the Results section but are missing in the following sections.*

There would be too many statistical results in the last sections, so in order to read easier the results of the statistical tests are placed into Table 1, S1 FIG, and S1 Table.

*Discussion
“Zebrafish have diurnal activity, meaning they are more active in the morning and less active at night…”. I think that the term “diurnal” refers to active periods during the day, not only in the morning.*

We appreciate the comment and corrected the sentence in the following way: “Zebrafish have diurnal activity, meaning they are more active during the day and less active at night.”

**Reviewer #2:**

*The paper entitled “A comparative study to optimize experimental conditions of pentylenetetrazol and pilocarpine-induced epilepsy in zebrafish larvae” by David Szep et.al. describes a series of experiments finalized to the optimization and standardization of the zebrafish larvae epilepsy models of Pentylenetetrazol and pilocarpine to support the development of new anti-epileptic drugs.
The authors describe the steps applied to execute the experiments, how they have analyzed the data and how their findings support their recommendations. The figures are generally well described and easy to understand. Moreover, the recommended method provides an easy readout, the larvae movement, it is a high-throughput technique and a replacement method since it uses not independently feeding larval forms.*

*Line 11: the authors report; ““Until 5 dpf larvae is not considered an animal”. According to the Directive 2010/63/EU the law is applicable on the independently feeding larval forms. The housing/rising temperature should also be reported since the age at which zebrafish larvae become independent feeding forms depends on this parameter.*

Thank you for your suggestion. The larvae that we used for the experiments did not reach the independent feeding form. To be clear we corrected the sentence in the introduction as the following: “As zebrafish larva starts to feed at 5 days post fertilization (dpf) and according to regulations the nonfeeding larva is not considered an animal, thus ethical permit is not required for performing experiments on ≤ 5dpf zebrafish larvae. Hence zebrafish larvae can be considered as a non-animal in vivo vertebrate model.”

*Materials and methods
The authors should also report in the material and methods:
• the zebrafish strain that they have used to generate the embryos;
• the housing condition of the zebrafish
• the mating system to obtain the zebrafish embryos;
• the incubation/housing conditions of the larvae before the experiments.
Line 3: the authors should also report the recipe of the egg water or quote a publication where is reported;
Line 4: the authors should state in which solvent the drugs are diluted.
Line 6: the authors should add the word “minutes” after “the solution for 30”
Line 9: the authors should write the accommodation time as reported in the Figure 1.*

*Line 9: “90 fish were moved into a 96 well plate”. The authors should specify if they have placed 30 control, 30 PTZ and 30 pilocarpine treated larvae in each 96 well plate. It is not stated in the manuscript.
Line 19: “we set up a 0.2 % threshold and excluded the remaining data”. The authors should explain what they have done.
Line 25: the authors should describe which method they have used to detect the outlier and what they have considered as an outlier.*

We extended the Material and methods part with the missing information.

*Line 26: The authors should consider the use of the Kruskall Wallis test when comparing more than 2 independent groups at the same time (e.g. Fig 4). Mann Whitney U test is suitable for comparing 2 independent groups.*

We indeed performed Kruskall-Wallis tests during the initial analysis which resulted in very similar results to Mann-Whitney U test. K-W test shows us that there is at least one group that is different from the others, but we were more interested in which one is different. We had the option to use Dunn’s post hoc test along with the K-W test or the M-W U test alone. We chose the M-W U test because in it is more straightforward and gave identical statistical significance to K-W.

*Results
Fig. 4: The authors should state the origin of the data reported in the Figure 4.*

To create the figures, we used our experimental data. In Figure 4, the significant differences were the result of the Mann-Whitney U test. We extended the figure legends with the name of the test we used.

*Fig. 6: There are 8 white bars instead of 9 bars representing the value for each trial.*

Thank you for your observation, one row of data was missing, we corrected it in the revised manuscript.

*Line 34-38:* *“We also performed a linear regression analysis with the cumulative values of the distance moved during the time intervals to assess if longer observation time could impact the difference between the groups. As seen in Fig 7 by increasing the time, the distances changed proportionally within the groups. This was supported by the linear regression analysis in most of the cases according to the R2 values (S2 Fig).”*

*The authors to support this statement should assess the possible difference among the regression coefficients (β) of the regression lines calculated for the control, the PTZ and the Pilocarpine groups through a hypothesis test.
Moreover, to support the above statement, the authors should also add a figure showing the regression lines calculated for the control, the PTZ and the Pilocarpine groups.*

Thank you for your suggestion, we created a new figure (Figure 8) showing the regression lines, among the missing regression coefficients (β) values.

*Discussion
Line 50. “We suggest that the recording algorithm has some feature that makes the first 5 minutes of recording unreliable, and thus needs to be disregarded.”
The author should double check with the recording algorithm maker.*

We indeed contacted the software developer company when we first observed this peculiar problem. After several lengthy discussions, they were unable to identify the reason for this reproducible observation. Since we are not in a position to find the real cause, we left the text in the manuscript as an observation and recommendation to avoid errors in the analysis without an explanation for the observation.

Finally, we would like to thank the reviewers and editors for evaluating our manuscript. We corrected all errors and followed the reviewers’ suggestions which certainly greatly improved the quality of the manuscript.
